# Elective adhesiolysis for chronic abdominal pain reduces long-term risk of adhesive small bowel obstruction

**DOI:** 10.1186/s13017-023-00477-9

**Published:** 2023-01-23

**Authors:** Barend A. W. van den Beukel, Masja K. Toneman, Fleur van Veelen, Marjolein Blusse van Oud-Alblas, Koen van Dongen, Martijn W. J. Stommel, Harry van Goor, Richard P. G. ten Broek

**Affiliations:** 1 Department of Surgery, Radboud University Nijmegen Medical Center, P.O. Box 9101, 6500 HB Nijmegen, The Netherlands; 2 Department of Surgery, Pantein Hospital Boxmeer, Beugen, The Netherlands

**Keywords:** Adhesive small bowel obstruction, Adhesiolysis, Recurrent small bowel obstruction, Chronic abdominal pain, Adhesion barrier, CineMRI

## Abstract

**Background:**

Selected patients with adhesion-related chronic abdominal pain can be treated effectively by adhesiolysis with the application of adhesion barriers. These patients might also have an increased risk to develop adhesive small bowel obstruction (ASBO). It is unknown how frequently these patients develop ASBO, and how elective adhesiolysis for pain impacts the risk of ASBO.

**Methods:**

Patients with adhesion-related chronic pain were included in this cohort study with long-term follow-up. The diagnosis of adhesions was confirmed using CineMRI. The decision for operative treatment of adhesions was made by shared agreement based on the correlation of complaints with CineMRI findings. The primary outcome was the 5-years incidence of readmission for ASBO. Incidence was compared between patients with elective adhesiolysis and those treated non-operatively and between patients with and without previous ASBO. Univariable and multivariable Cox regression analysis was performed to identify predictive factors for ASBO. Secondary outcomes included reoperation for ASBO and self-reported pain and other abdominal symptoms.

**Results:**

A total of 122 patients were included, 69 patients underwent elective adhesiolysis. Thirty patients in both groups had previous episodes of ASBO in history. During 5-year follow-up, the readmission rate for ASBO was 6.5% after elective adhesiolysis compared to 26.9% after non-operative treatment (*p* = 0.012). These percentages were 13.3% compared to 40% in the subgroup of patients with previous episodes of ASBO (*p* = 0.039). In multivariable analysis, elective adhesiolysis was associated with a decreased risk of readmission for ASBO with an odds ratio of 0.21 (95% CI 0.07–0.65), the risk was increased in patients with previous episodes with a odds ratio of 19.2 (95% CI 2.5–144.4). There was no difference between the groups in the prevalence of self-reported abdominal pain. However, in surgically treated patients the impact of pain on daily activities was lower, and the incidence of other symptoms was lower.

**Conclusion:**

More than one in four patients with chronic adhesion-related pain develop episodes of ASBO when treated non-operatively. Elective adhesiolysis reduces the incidence of ASBO in patients with chronic adhesion-related symptoms, both in patients with and without previous episodes of ASBO in history.

*Trial registration* The study was registered at Clinicaltrials.gov under NCT01236625.

**Supplementary Information:**

The online version contains supplementary material available at 10.1186/s13017-023-00477-9.

## Introduction

Adhesions are internal scars that develop in 90% of patients undergoing open abdominal surgery, and 70% of patients undergoing laparoscopy [1, 2]. Adhesions can cause a wide range of sequelae, such as chronic abdominal pain and adhesive small bowel obstruction (ASBO) [3, 4]. Our university hospital is a referral center for patients with adhesion-related abdominal symptoms, mostly chronic pain and recurrent bowel obstruction. Part of these patients is operatively treated by elective adhesiolysis with adhesion barriers, as selected by shared-decision making based on patient preferences and results of noninvasive mapping of adhesions by CineMRI. We have previously demonstrated favorable pain results of this approach, with long-term improvement of pain in 80% of patients [5].

Patients with chronic adhesion-related pain might also have an increased risk to develop ASBO. Previous studies reported that patients with adhesion-related pain often have additional gastrointestinal symptoms including nausea, bloating, and difficulty with stools [3, 6, 7]. In an analysis of CT findings, Gopireddy et al. described that dilation and contortion of the bowel might be seen in patients with adhesions without an acute obstruction [8]. Conversely, patients after operative treatment for ASBO have a 20% risk of developing chronic pain [9]. These findings suggest that ASBO and chronic abdominal pain might be interrelated. From our experience, particularly patients with a history of ASBO ask questions relating to the effect of adhesiolysis on the risk of a new episode of ASBO in addition to the outcome of pain.

There is increasing circumstantial evidence that elective adhesiolysis for recurrent ASBO reduces the risk for new episodes of ASBO in comparison with non-operative management or natural course [10–12]. Small cohort studies have demonstrated a relatively low risk of future episodes of ASBO following laparoscopic adhesiolysis in patients with recurrent episodes [12–15]. These series, however, lack a control group in which the natural course of recurrence was evaluated. On the other hand, even partial reformation of adhesion might undo the benefits of surgery to reduce the risk of ASBO and ASBO can even be caused by just one strand of adhesions [16]. The use of an adhesion barrier has not been properly studied in elective adhesiolysis for recurrent ASBO. In a trial of emergency open surgery for ASBO, a liquid adhesion barrier lowered the recurrence risk compared with no barrier [17].

This study aims to assess the long-term incidence of readmission for new episodes of ASBO in patients with chronic symptoms of adhesions (pain or recurrent obstruction), comparing patients who underwent elective adhesiolysis plus a barrier to those treated non-operatively and comparing patients with and without previous episodes of ASBO. We also aim to identify factors that predict the occurrence of ASBO. Results will provide a better insight into the risk of devlopping ASBO in patients with adhesion-related chronic pain and recurrent small bowel obstruction, and how this risk is affected by elective adhesiolysis. Such insight may improve the management of patients with chronic abdominal symptoms related to adhesions.

## Material and methods

### Study design and patients

This study is a longitudinal follow-up study expanding on a previous prospective cohort (registered at clinicaltrials.gov under NCT01236625) of patients with chronic adhesion-related abdominal pain [5]. All patients referred to the outpatient clinic of the Radboud University Medical Center in Nijmegen, the Netherlands, from January 2012 to December 2019 for evaluation of adhesion-related pain were eligible. Patients were primarily referred for chronic abdominal pain, some also presented with recurrent episodes of small bowel obstruction or a combination of both. Patients were included if the diagnosis of adhesions was confirmed on CineMRI.

All included patients had a minimum of 2 years of follow-up. Exclusion criteria were age below 18 years, mental incompetence, and absence of adhesions on CineMRI. All patients gave written informed consent for participation in the study. A waiver was obtained from the medical ethical committee of the Radboud University Medical center (registration number: 2021/7398) for this study, according to Dutch law. The study was conducted in accordance with the principles of the revised version of the Declaration of Helsinki (2013, Fortaleza). Data were analyzed anonymously.

For this study, we grouped patients according to their initial treatment. The first group consisted of patients who had undergone elective adhesiolysis with the use of an adhesion barrier (operative group). Patients in the second group were non-operatively treated receiving only conservative symptomatic treatments. The decision on whether or not to perform adhesiolysis was made through shared-decision making, incorporating the results of CineMRI (localization and extent of adhesions), individualized operative risks and benefits (comorbidities, number of previous laparotomies), and patient preferences [5]. Adhesiolysis was either performed by laparoscopy or open approach, depending on safe abdominal entry considerations and the patient’s (and surgeon’s) preference.

In laparoscopic completed adhesiolysis, the liquid icodextrin 4% (Adept®) anti-adhesion barrier was applied. A 1500 ml infusion bag of icodextrin 4% was applied using the laparoscopic irrigator. The first 500 to 800 ml were used for rinsing and to remove remaining blood cloths, leaving between 700 to 1,000 milliliters of barrier fluid to be installed at closure of the abdomen. During converted or open adhesiolysis, in addition to icodextrin 4%, sheets of hyaluronate carboxymethylcellulose (Seprafilm®) were applied on large regular shaped surfaces, such as between bowel and omentum, or bowel and ventral, lateral of posterior abdominal wall. Typically, between three to six hyaluronate carboxymethylcellulose sheets measuring 7.5 by 13 cm were used.

All included patients were contacted to answer a questionnaire regarding current abdominal symptoms focusing on adhesive small bowel obstruction, analgesic medication, and healthcare utilization. Further, they were asked for permission to send queries to obtain medical data from their general practitioners and local hospitals. This was deemed necessary because the Radboud University Medical Center is a tertiary referral center and new cases of ASBO might have been treated in a local hospital.

### Data collection

To identify potential readmissions for ASBO, we reviewed the results from the patient questionnaires and general practitioners. In the Netherlands, all patients are assigned to a general practitioner who keeps a full medical record and receives hospital correspondence from any admission by any hospital [18, 19]. Therefore, the general practitioner record is a reliable source to identify readmissions. When a potential readmission for ASBO in a different hospital was identified, additional data was requested from that hospital to confirm the diagnosis and gather data on treatment and outcomes.

Data collected from medical records included age and sex, number of previous abdominal surgeries, abdominal or pelvic radiotherapy in history, number of ASBO episodes in history, previous adhesiolysis for ASBO, and the extent of adhesions described on CineMRI for both groups. The extent of adhesions on CineMRI and the extent of adhesions assessed during adhesiolysis was graded on a 5-point scale (0 = no adhesions, 1 = single strand, 2 = adhesions in one quadrant, 3 = adhesions in two quadrants and 4 = adhesions in three or four quadrants). In the group undergoing elective adhesiolysis, we assessed surgical approach (open or laparoscopic), conversion, inadvertent bowel injury, length of hospital stay, complications graded according to the Clavien–Dindo classification, and admission to the ICU [20].

### Questionnaire

The project steering group conceived the first set of multiple-choice survey questions. These questions were edited by two independent researchers, with experience in surveys and questionnaire research. The questionnaire was subsequently tested for clarity and ease of use by a group of laymen. After processing the feedback on our questionnaire, the final version was conceived.

The questionnaire consisted of eight required multiple-choice questions and 19 dependent questions. Further, a free text field for clarifications and personal comments was included. The questionnaire collected data regarding readmission for small bowel obstruction, and gastrointestinal symptoms pointing at motility disturbances such as nausea, vomiting, and difficulties with stools. Further, we screened for pain disability and medical consumption. An English translation of the questionnaire can be found in Online Additional file 1.

The questionnaire data were managed using Castor software (Ciwit, Amsterdam, The Netherlands) which has been optimized for data capture in medical research according to good clinical practice standards. The questionnaires were directly collected using a secured link sent with Castor or in a paper version, depending on the patient’s preference and digital literacy. The questionnaire was open for three months. Reminders were sent out after one month and two months. Participants were also contacted by phone in conjunction with a second reminder.

### Data analysis

The primary outcome of this study was the incidence of a new episode of ASBO compared between the operative and non-operative groups. Subgroup analysis was performed for patients with and without a previous episode of ASBO. Multivariable analysis was performed to correct for baseline differences and to identify independent risk factors for developing ASBO.

Main secondary outcome was the incidence of reoperation for ASBO. Additional outcomes were results of ASBO treatment, daily gastrointestinal symptoms related to obstruction such as nausea, vomiting, daily pain, use of medication, and visits to medical specialists or other healthcare providers. Secondary outcomes were descriptively analyzed.

The incidence of ASBO was analyzed using 1-survival Kaplan–Meier methods. Comparison between groups was made using Cox regression analysis, and a *p*-value < 0.05 was considered significant.

Categorical data were analyzed using a Chi-square or Fisher’s exact test, as appropriate. Continuous data were analyzed using an independent t test or Mann–Whitney U test if not normally distributed. Continuous variables are presented as means with standard deviation, or medians and range in case of non-normal distribution. Dichotomous or categorical variables are presented as absolute numbers and percentages. All analyses are performed using SPSS version 28°0 (Armonk, NY: IBM Corp). Univariable and multivariable Cox regression analyses were performed to identify factors that independently affect the incidence of readmission for ASBO. Predictive factors with *p* ≤ 0.20 in univariable were selected as candidate risk factors for multivariable analysis. In multivariable analysis, a stepwise backward selection procedure was used with a *P*-entry ≤ 0.20 and *P*-stay ≤ 0.10. Potential predictors for readmissions for ASBO we used in univariable analysis were sex, the number of abdominal surgeries, ASBO in history, number of ASBO in history, the extension of adhesion on CineMRI, treatment of adhesions (operative vs. non-operative), and type of surgery during elective adhesiolysis (laparoscopy, converted, open).

## Results

**Fig. 1:**
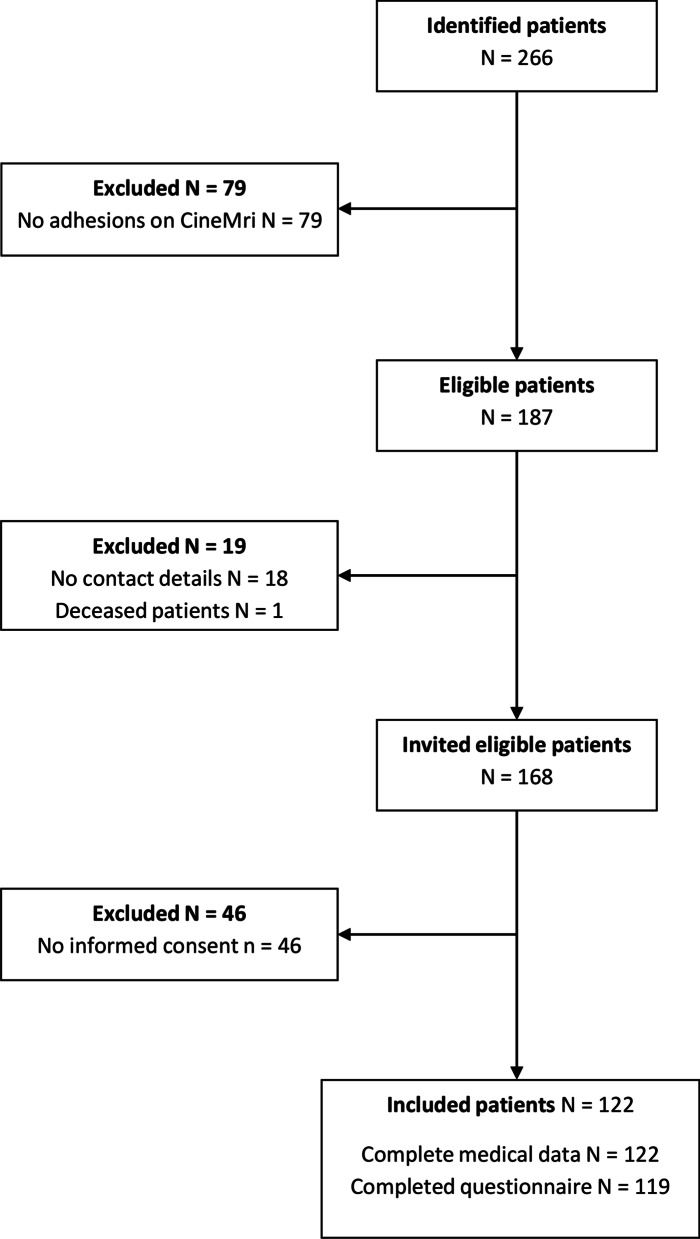
Flowchart patient selection

A total of 266 patients referred to the outpatient clinic of the Radboud University Medical Centre underwent a CineMRI between 2012 and 2019 for evaluation of chronic abdominal pain possibly attributed to postoperative adhesions. Seventy-nine patients had no adhesions on the CineMRI leaving 187 patients eligible for inclusion. No contact details were available for 18 patients, and one patient died from an unrelated cause. A total of 122 of the remaining 168 patients (72.6%) provided informed consent to participate in the study (Fig. 1). Medical data could be obtained from all 122 participants, and 119 patients returned a completed questionnaire.

### Baseline characteristics

Participants and non-responders were comparable in baseline characteristics, except that the percentage of patients with a history of ASBO was higher among participants (49.2% as compared to 29% in non-responders [*p* = 0.009]). Among non-responders, the number of patients with a previous episode(s) of ASBO was equally distributed between the operative and non-operative groups [Online Additional file 1].

Sixty-nine of 122 participants (57%) underwent elective adhesiolysis. Fifty patients (72.5%) underwent laparoscopic adhesiolysis of whom 17 (34%) were converted to laparotomy. In 67 (97.1%) patients a barrier was used for prevention of adhesion reformation. In two patients, a barrier was not placed, in one because of gross contamination of the abdomen, and in the other because the appropriate barrier was not available. An iatrogenic enterotomy was reported in six patients (8.7%) and treated with primary closure. Postoperative complications were reported in 14 patients of whom four (5.6%) patients had a severe complication graded as Clavien–Dindo score 3. These four patients (5.6%) underwent a relaparotomy. During relaparotomy, an iatrogenic injury was found in two patients. The other two patients underwent diagnostic laparotomy for severe postoperative pain and strongly elevated inflammatory markers with no abnormalities found. The median length of hospital stay (LOS) was four days (range 1–49), no patients were admitted to the intensive care unit during admission, and there was no mortality.

The operative and non-operative groups were comparable in baseline characteristics, with only minor differences (Table 1). Mean follow-up in the operative group was 70 ± 27 months compared to 64 ± 23 months in the non-operative group (*p* = 0.245). In the operative group, 44 (60.3%) had a follow-up of 5-years or more, compared to 29 (54.7%) in the non-operative group (*p* = 0.312).

**Table 1:** Baseline characteristics

Factor	Operative, elective adhesiolysis	Non-operative	*p*-value
Number of patients	69	53	
Sex			0.548
Male	15 (21.7%)	14 (26.4%)	
Female	54 (78.3%)	39 (73.6%)	
Age in years*	50.2 ± 11.0	52.8 ± 12.7	0.435
Number of previous surgeries†	3 (1–8)	3 (1–44)	0.472
Radiotherapy in history	1 (1.4%)	3 (5.7%)	0.195
ASBO in history (N)	30 (43.5%)	30 (56.6%)	0.151
Number of ASBO episodes in history†	2 (0–11)	3.5 (0–25)	0.210
Conservative treated ASBO†	1 (0–10)	1.5 (0–25)	0.074
Surgical treated ASBO†	1 (0–5)	1 (0–5)	0.630
Extend of adhesions on CineMRI†	2 (1–4)	2 (1–4)	0.235
Length of follow-up in months*	70 ± 27	64 ± 23	0.245

*Mean ± SD, † median (range)

### Incidence of ASBO

**Fig. 2:**
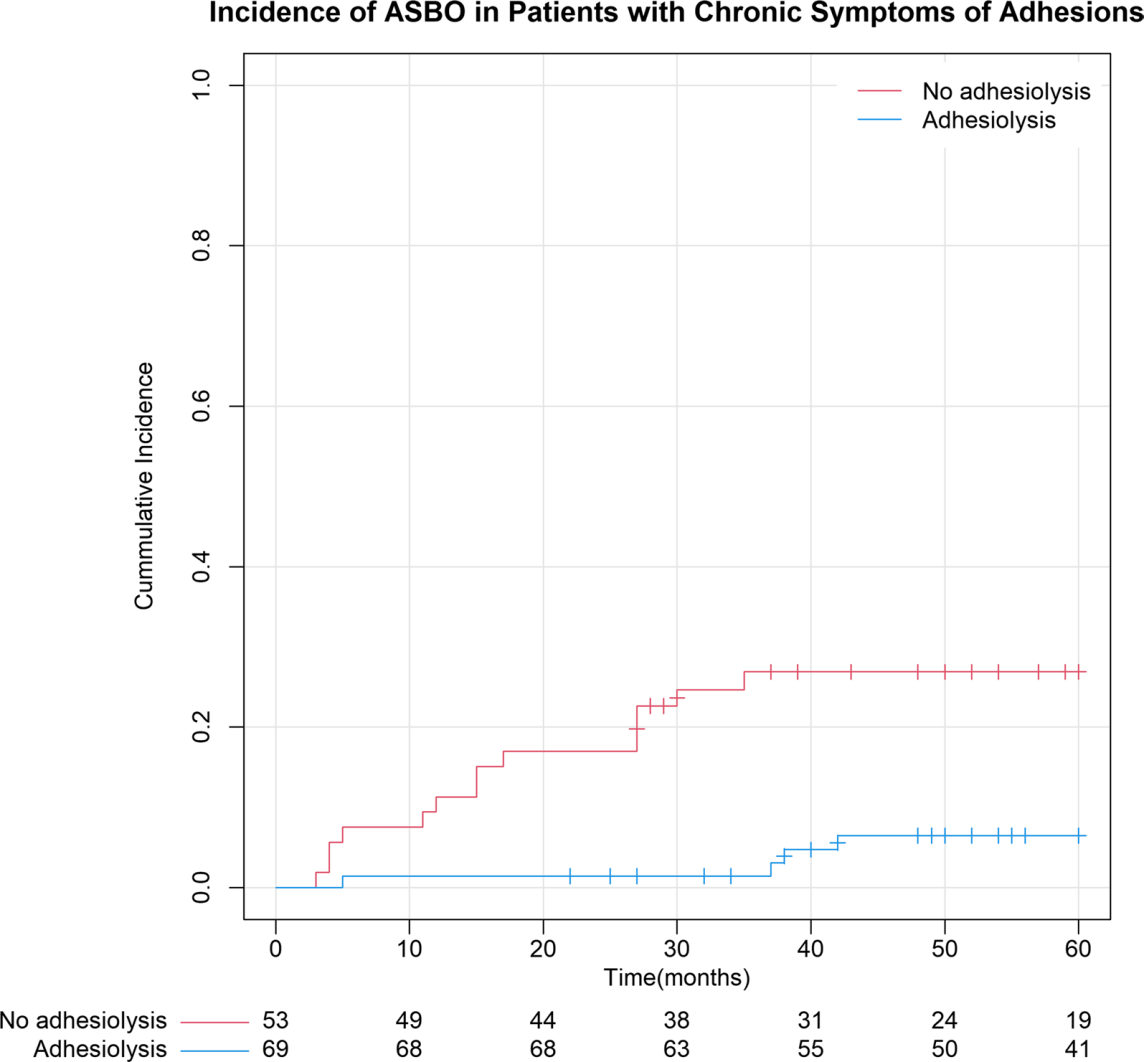
Incidence of ASBO 5-year survival

Operated patients had a significantly lower risk of developing new episodes of ASBO. The 5-year incidence of ASBO was 6.5% (95% CI: 0.2%-12.8%) following elective adhesiolysis compared with 26.9% (95% CI: 14.7–39.3) in the non-operative group, which difference was significant (*p* = 0.001). The median time to a new episode of ASBO was 37 (range 5–42) months compared to 15 (range 3–35) months (*p* < 0.001) (Fig. 2).

**Fig. 3:**
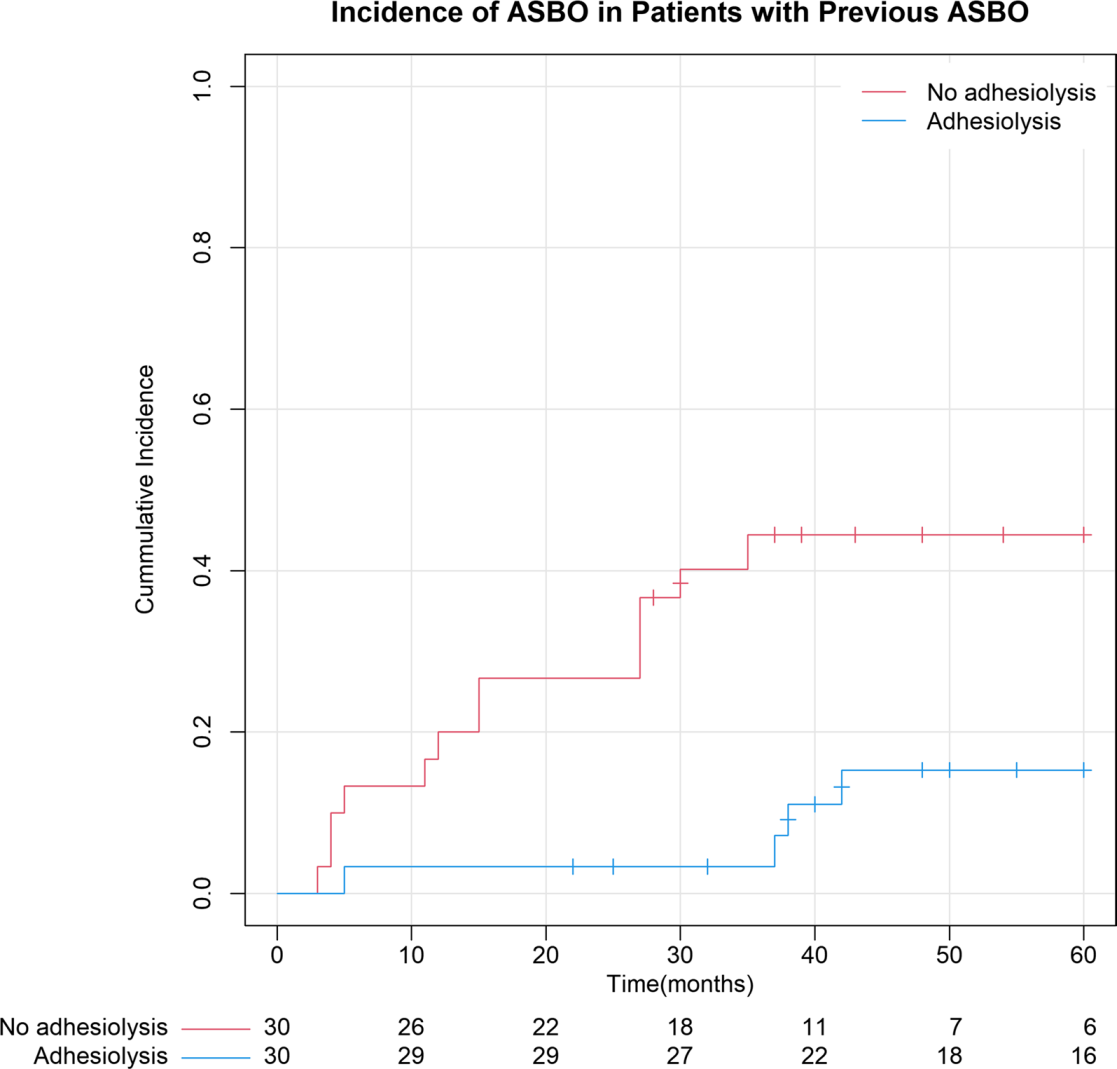
Incidence ASBO 5-year survival in patients with previous ASBO

In the subgroup of patients with a history of one or more episodes of ASBOs, the difference was also significant with a 5-year incidence of 15.3% (95% CI: 1.4–29.3%) following adhesiolysis compared with 44.5% (95% CI: 26.2- 62.7%) in the non-operative group (*p* = 0.005). Median time to a new episode of ASBO also differed between groups (37.5 (range 5–42) months compared to 15.0 (range 3–35) months, (*p* < 0.001) (Fig. 3).

### Predictors for ASBO

In univariable analysis, one or more previous episodes of ASBO in history, and the extent of adhesions on CineMRI were correlated with an increased risk of a new episode of ASBO. Operative treatment of chronic adhesion-related pain was correlated with a lower incidence of ASBO (Table 2 a).

**Table 2:** a. Univariable analysis for risk factors of new episodes of ASBO. b. Multivariable analysis

(a) Factor	ASBO (N, %)	OR	95% CI	P
*Demographics*				
Gender				
Male	5/29 (17.2%)	Ref		
Female	13/93 (14.0%)	0.78	0.28–2.20	0.643
Age* (each year increase)	52.7 (± 15.5)*	1.02	0.98–1.06	0.448
Readmission for ASBO	53.0 (± 15.0)			
No readmission for ASBO	51.1 (± 11.2)			
*Patient history*				
ASBO in history				
No	1/ 62 (1.6%)	Ref		
Yes	17/60 (28.3%)	20.8	2.80–156.4	0.003
Number of previous ASBO				
No previous ASBO	1/62 (1.6%)	Ref		
One episode of ASBO	2/19 (10.5%)	6.9	0.62–75.9	0.115
Multiple episodes of ASBO	15/41 (36.6%)	28.8	3.79–218.6	0.001
Treatment of previous ASBO				
No ASBO	2/63 (3.2%)	Ref		
Conservative	6/17 (35.3%)	14.8	2.97–73.2	0.001
Operative	10/42 (23.8%)	8.5	1.85–38.6	0.006
Number of previous surgery				
One surgical procedure	3/18 (16.7%)	Ref		
Multiple surgical procedures	15/104 (14.4%)	0.86	0.25–2.97	0.809
Type of previous surgery				
Laparoscopy	2/24 (8.3%)	Ref		
Laparotomy	16/98 (16.3%)	1.99	0.46–8.65	0.360
*Diagnostic results*				
Extent of adhesions on CineMRI				
Suspected single strand	2/32 (6.3%)	Ref		
One quadrant	11/52 (21.2%)	3.78	0.84–17.1	0.084
Two quadrants	3/25 (11.8%)	2.00	0.33–11.9	0.449
Three or four quadrants	2/13 (15.4%)	2.58	0.36–18.3	0.343
*Treatment of adhesion-related pain*				
Type of treatment				
Non-operative	14 (26.4%)	Ref		
Operative	4 (5.8%)	0.19	0.06–0.58	0.003
Type of surgery during elective adhesiolysis				
Laparoscopy	1/33 (3.0%)	Ref		
Conversion	1/17 (5.9%)	1.63	0.10–26.0	0.731
Laparotomy	2/19 (10.5%)	3.37	0.31–37.3	0.321

(b) Factor	OR	CI-95%	*p*-value
ASBO in history	19.2	2.54–144.4	0.004
Count of ASBO in history	NS	NS	NS
Treatment of ASBO	NS	NS	NS
Operative treatment (elective adhesiolysis)	0.21	0.07–0.65	0.007

*Mean and standard deviation of age

*NS* not selected for model in stepwise multivariable analysis

In multivariable analysis, previous ASBO was an independent risk factor for developing a new episode of ASBO with an odds ratio of 19.2 (95% CI: 2.54–144.4; *p* = 0.004). Operative treatment was an independent factor in decreasing the risk of a new episode of ASBO with a odds ratio of 0.21 (95% CI: 0.07–0.65; *p* = 0.007). All other factors were excluded from the multivariable analysis. (Table 2b).

### Secondary outcomes

In the operative group, five patients were readmitted for ASBO and in the non-operative group 14 patients were readmitted. All patients were readmitted within 60 months of follow-up, expect one patient in the operative group who was readmitted 8 years after elective adhesiolysis. Most patients (63%) who were readmitted for ASBO had two or more episodes within 60 months of follow-up. Four patients underwent an emergency operation for ASBO, one (1.4%) in the initial operative group and three (5.7%) in the initial non-operative group (*p* = 0.240). One complication of acute adhesiolysis was reported. This patient had an intra-abdominal abscess and prolonged postoperative ileus after adhesiolysis, which was treated by radiological drainage of the abscess. The patient was discharged after 22 days from the hospital.

### Questionnaire

The prevalence of abdominal pain was comparable between both groups. The effect of pain on daily activities tended to be lower in the operative group compared to the non-operative group (mean score 2.4 (± 2.0) and 3.2 (± 1.6), *p* = 0.05). In the operative group, 17 (25.8%) patients reported persistent abdominal symptoms other than pain compared to 24 (45.3%) in the non-operative group, *p* = 0.017.

In the operative group, 7 (10.1%) patients consulted a surgeon during follow-up compared to 13 (24.5%) patients in the non-operative group (*p* = 0.033). Psychologists were also less frequently consulted, one (1.5%) patient compared to five (9.4%) patients, *p* = 0.05. Visits to the general practitioner and other medical specialists were comparable between groups.

## Discussion

This study was designed to assess the effect of elective surgery with adhesiolysis in patients with chronic adhesion-related pain on the long-term incidence of ASBO, predictive factors of readmission of ASBO and patient-reported abdominal symptoms. Adhesiolysis with an anti-adhesive barrier reduced the occurrence of ASBO by a factor four to 6.5 percent in the whole cohort and by a factor three to 15 percent in patients with one or more previous episodes of ASBO. In comparison, the incidence of ASBO is 2% in patients after any abdominal surgery and 10% in patients after colorectal surgery [4, 21]. We found no other risk factors than adhesiolysis and previous ASBO. Elective adhesiolysis reduced most patient-reported long-term chronic abdominal symptoms and the use of healthcare services. Altogether, elective adhesiolysis with barrier use for chronic abdominal pain has a broader impact on a patient’s life than just long-term pain relief.

The topic of elective adhesiolysis has been surrounded by some controversies, regarding its effectiveness, indications, surgical technique, and safety. The main body of literature on elective adhesiolysis addresses chronic adhesion-related pain, with several additional case series reporting on elective adhesiolysis for recurrent ASBO [12, 13, 15]. Most studies reporting on adhesiolysis for chronic pain reported a good initial, short-term, response in 70–80% of patients [22]. In a trial randomizing between diagnostic laparoscopy and adhesiolysis, the recurrence of symptoms at one year was high in both groups [23]. However, no barriers were used in this trial. In a recent study from our group, 80% of patients reported improvement in pain after 1.5 years following elective adhesiolysis with a barrier [5]. Also in a trial by Cheong et al. adhesiolysis with a barrier resulted in significant improvement of pain at 6 months, as compared to diagnostic laparoscopy [24]. Thus, recent studies suggest that adhesiolysis with barriers can be effective in selected patients reducing chronic pain and improving quality of life.

The lowered occurrence rate of ASBO following elective surgery with adhesiolysis and the application of a barrier add to the potential benefits of this procedure. ASBO is an acute surgical condition that is usually considered resolved after treatment. However, ASBO inherits a high risk of recurrence and can also impact the long-term quality of life [11, 12, 25]. Recurrence risk following conservative treatment is 21–25% and following operative management 13–19% in the first ten years after the first episode of ASBO [10, 11]. With every new episode of ASBO, the risk of recurrent episodes increases and the interval between episodes decreases, resulting in a considerable number of patients developing rapidly recurring episodes of ASBO [26]. Some patients with recurrent ASBO end up on liquid or low-residue diets to prevent new episodes of readmission [27]. Question remains whether or not elective adhesiolysis should be considered for patients presenting with recurrent ASBO as the main symptom. Two small case series reported some favorable results performing elective adhesiolysis for this indication [12, 14]. In these series, up to one in eight patients developed a recurrent episode of ASBO during four to five years of follow-up following elective adhesiolysis. Patients had a median of two to three previous episodes of ASBO. These studies lacked a control group; nevertheless, these results seem to compare favorably to the 30–60% 10-year recurrence rates reported in the natural course of patients with multiple episodes of ASBO in history [28]. In our study, the risk of recurrent ASBO in the subgroup of patients with previous ASBO was considerably lower after adhesiolysis when compared to the non-operative group; the latter was at a comparable risk for developing recurrent episodes as expected from reports on natural course. In both our cohort and previous series reporting on elective adhesiolysis, most patients had less than four episodes prior to surgery. Epidemiological data show that with more episodes the risk of recurrence increases with a shortening interval between episodes. In patients with a high frequency of recurrence, new episodes might therefore be more difficult to prevent [28, 29].

A benefit of performing adhesiolysis as elective surgery and not as an emergency procedure to reduce the risk of recurrent ASBO is the lower risk of iatrogenic injuries and mortality. In the NELA report, 30-day mortality associated with emergency adhesiolysis was 5%, which increased to 11.8% in high-risk patients with factors such as frailty, elderly, or comorbidities [30, 31]. In the emergency setting, patients are admitted in a deteriorated condition. The distended bowel in the emergency setting increases the risk of conversion and injuries during surgical handling [32, 33]. In contrast, elective adhesiolysis can be considered a safer procedure. A few bowel injuries were found which healed uneventfully mostly after simple primary closure and there were no postoperative deaths.

Although we included a relatively large number of patients undergoing elective adhesiolysis, a limitation of this study is the relatively small size of different subgroups. Results on treatment and outcome of new episodes of ASBO should therefore be interpreted with caution. Readmission for ASBO is a commonly used outcome for follow-up studies on ASBO and is easy to establish objectively. Nevertheless, in patients with frequent episodes, mild cases of obstructive symptoms might not always result in admission. Another potential limitation is selection bias, as patients were allocated to treatment shared-decision making and not by randomization. Potentially, patients with more extensive adhesions on CineMRI imaging who are at an increased risk of iatrogenic injuries during adhesiolysis might have been more prone to be allocated to the non-operative group, resulting in more favorable results for the operative group. However, from our experience and review of patient files, extensive adhesions were only seldom the main reason to choose non-operative treatment. In most cases, other patient factors or patient preferences were the decisive factors for treatment decisions. The extent of adhesions also seemed evenly distributed among groups at baseline comparison.

Given the high risk of ASBO in patients with chronic adhesion-related pain, reducing the risk of ASBO can be an additional benefit when considering operative treatment. Results also seem promising for patients presenting with recurrent small bowel obstruction, with or without chronic pain. Data for patients with a high frequency of recurrence (i.e., 4 episodes or more) of ASBO, however, remain limited. Further, it is not known if the results also apply to patients with abdominal radiotherapy in history, who often have more extensive adhesions. The potential benefits of reducing the risk of recurrence should be weighed against the risks of an operation. Although adhesiolysis is associated with some considerable risk, and iatrogenic bowel injury, in particular, there is also growing evidence for the important impact of preventing recurrences of ASBO. In a recent analysis, Behman. et al. demonstrated an improved long-term survival of ASBO after emergency operative treatment as compared to conservative treatment, mediated by a lower risk of recurrence. [10] The lower operative risks of elective adhesiolysis might contribute to an even greater benefit.

## Conclusion

More than one in four patients with chronic adhesion-related pain develop episodes of ASBO when left untreated. Elective adhesiolysis reduces the incidence of ASBO in patients with chronic adhesion-related pain, both in patients with and without previous episodes of ASBO. The results implicate that elective adhesiolysis could improve long-term QoL in patients with chronic adhesion-related symptoms, both by reducing pain and the risk of ASBO. The results also seem promising for patients primarily presenting with recurrent ASBO, with or without chronic pain.

## Supplementary Information

**Additional file 1.** English tranlation of Questionnaire and Baseline Characteristics of Non-Responders.

## Data Availability

The datasets used during the current study are available from the corresponding author on reasonable request.
