# Supplementary material for: Elective adhesiolysis for chronic abdominal pain reduces long-term risk of adhesive small bowel obstruction
Source: World J Emerg Surg. 2023 Jan 23;18:8. doi: 10.1186/s13017-023-00477-9 (PMC9872389; doi:10.1186/s13017-023-00477-9)
Supplement: Supplementary file 1 — Additional file 1. English tranlation of Questionnaire and Baseline Characteristics of Non-Responders. [file 13017_2023_477_MOESM1_ESM.docx]

**Appendix A**

**Table of contents:**

**Part 1. Questionnaire
Part 2. Characteristics of non-responders**

**Part 1. Questionnaire**

**Questionnaire Abdominal Pain following diagnosis of Adhesions**

1. After the diagnosis of adhesions, did you suffer from complaints that could possibly be related to an intestinal obstruction? (I.e.. nausea, vomiting, distended abdomen, constipation)
2. Nausea
   - Never
   - Rarely
   - Occasionally
   - Often
3. Vomiting
   - Never
   - Rarely
   - Occasionally
   - Often
4. Distended abdomen
   - Never
   - Rarely
   - Occasionally
   - Often
5. Constipation
   - Never
   - Rarely
   - Occasionally
   - Often
6. After the diagnosis of adhesions, were you admitted to the hospital for an intestinal obstruction (Adhesive small bowel obstruction, ileus)?
7. At least once:

- Yes
- No

1. If yes, how many times? _____
2. If yes, how was the obstruction treated?
   - Every time without surgery
   - One or multiple times by surgery
3. Did your abdominal complaints change in the period after the diagnosis of adhesions?
   - Very much improved
   - Improved
   - Minimally improved
   - Same as before the diagnosis
   - Minimally worsened
   - Worsened
   - Very much worsened
4. Encircle the number that best fits your pain intensity at this moment±

0 1 2 3 4 5 6 7 8 9 10

No pain Worst imaginable pain

1. How often do you experience abdominal pain complaints?

- Rarely to never
- Now and then
- Often
- Continuously

1. To what extent are you hindered by abdominal pain in your daily activities?
   - Not at all
   - A little bit of hinder
   - Quite some hinder
   - Much hinder
   - Invalidating hinder
2. Which health care providers did you visit for abdominal pain, after the diagnosis of adhesions?
   - General practitioner
   - Pain expert or anaesthesiologist
   - Gynaecologist
   - Surgeon
   - Gastroenterologist
   - Emergency department
3. Which paramedics or therapists did you visit for abdominal pain, after the diagnosis of adhesions?
   - Physiotherapist
   - Psychologist
   - Osteopath
   - Acupuncturist
   - Other: ______
4. Do you use medications abdominal pain?

________________

1. Other comments.

________________

**Part 2. Characteristics of non-responders**

| ***Non-responders*** | | | |
| --- | --- | --- | --- |
| **Factor** | **Operative treatment** | **Non-operative treatment** | **p-value** |
| Number of patients | N= 32 | N= 30 |  |
| Sex |  |  | 0.040 |
| Male | 10 (31.2%) | 3 (10.0%) |  |
| Female | 22 (68.8%) | 27 (90.0%) |  |
| Age | 49.5 (±16.1) | 55.3 (±13.2) | 0.127 |
| ASBO in history | 9 (28.1%) | 9 (30%) | 0.871 |
| Number of ASBO (mean ±SD) | 0.5 (±1.1) | 0.55 (±1.0) | 0.846 |
| Number of previous surgery | 2.5 (±1,5) | 3.7 (±2.8) | 0.041 |
| Extent of adhesions on CineMRI | 2.3 ±1.0 | 2.7 (±0.7) | 0.052 |
| Extent of adhesions peroperative | 2.81 (±0.9) |  |  |
| Zühlke classification | 2.7 (±0.9) |  |  |
| Laparoscopy | 30 (48.4%) |  |  |
| Conversion | 2 (3.2%) |  |  |
| Laparotomy | 24 (38.7%) |  |  |
| Iatrogenic injury | 2 (3.2%) |  |  |
| Complications | 4 (6.5%) |  |  |
| Relaparotomy | 2 (3.2%) |  |  |
| Length of hospital stay |  |  | 0.002 |
| Laparoscopy | 4 (1-13) |  |  |
| Laparotomy | 9 (1-25) |  |  |

| **Baseline characteristics of participants vs non-responders** | | | |
| --- | --- | --- | --- |
| **General** | **Participants** | **Non-responders** | **P-value** |
| Number of patients | 122 (100%) | 62 (100%) |  |
| Operative treatment | 69 (56.6%) | 32 (51.6%) | 0.315 |
| Non-operative treatment | 53 (43.4%) | 30 (48.4%) | 0.315 |
| Gender |  |  | 0.408 |
| Male | 29 (23.8%) | 13 (21.0%) |  |
| Female | 93 (76.2%) | 49 (79%) |  |
| Age | 51.4 (±11.8) | 52.3 (±14.9) | 0.672 |
| ASBO in history | 60 (49.2%) | 18 (40,9%) | 0.009 |
| Number of ASBO | 3.8 (±4.3) | 1.78 (±1.2) | 0.004 |
| Number of previous surgery | 3.7 (±4.3) | 3.1 (±2.3) | 0.305 |
| Extent of adhesions on CineMRI | 2.2 (±0.9) | 2.5 (±0.9) | 0.037 |
| **Adhesiolysis** | n=53 | n=32 |  |
| Extent of adhesions peroperative | 2.9 (±0.8) | 2.81 (±0.9) | 0.548 |
| Zühlke classification | 2.9 (±0.7) | 2.7 (±0.9) | 0.345 |
| Surgical procedure |  |  | 0.000 |
| Laparoscopy | 50 (72.5%) | 24 (75.0%) |  |
| Conversion | *17 (34%)* | 0 (0.0%) |  |
| Laparotomy | 19 (27.5%) | 7 (25%) |  |
| Iatrogenic injury | 19 (27.5%) | 2 (7.1%) | 0.027 |
| Complications | 14 (20.3%) | 4 (12.5%) | 0.610 |
| Relaparotomy | 4 (5.8%) | 2 (6.7%) | 0.610 |
| Length of hospital stay |  |  | 0.943 |
| Laparoscopy | 3 (1-11) | 4 (1-13) |  |
| Conversion | 6 (3-15) | 0 (NA) |  |
| Laparotomy | 9 (1-49) | 9 (1-25) |  |
